# Social environment of people living with dementia: Characteristics, facilitators and barriers

**DOI:** 10.1002/alz.087923

**Published:** 2025-01-09

**Authors:** Hanna Lea Knecht, Francisca S Rodriguez

**Affiliations:** ^1^ German Center for Neurodegenerative Diseases e.V., Greifswald, Mecklenburg‐Vorpommern Germany; ^2^ German Center for Neurodegenerative Diseases (DZNE), site Rostock/Greifswald, Greifswald, Mecklenburg‐Vorpommern Germany

## Abstract

**Background:**

The number of people with dementia increases worldwide. Previous studies have shown that social integration and high‐quality social relationships are beneficial for reducing dementia risk and improving symptoms. Our project aimed at identifying characteristics of the social environment of people with dementia (PWD) and their relevance for PWDs’ wellbeing, and at determining facilitators and barriers of the PWD’s social integration.

**Method:**

A total of N = 497 participants (86% female, mean age of 53 years, 53% urban), of whom 3% had dementia, 16% cared for family members, 17% cared for someone outside of family, 15% were professional caregivers, 16% were counsellors, and 33% were otherwise connected to dementia care, answered our survey questions. We performed descriptive data analyses.

**Result:**

The majority of participants (72%) reported that those caring for PWD initiated social interactions and more than half of the participants (57%) showed that these interactions are often experienced at home. Most participants (85%) felt that simply spending time with the PWD has a great positive impact on their well‐being. Partners of the PWD were considered as the most important social contact to reduce fears (score 4.8/5), to support them in managing everyday life (score 4.6/5), and to contribute to general well‐being (score 4.6/5). Regarding facilitators, most participants (89%) stated that family members are crucial for undertaking social activities. All participants (100%) felt that meeting face‐to‐face is necessary to maintain a relationship with a PWD and indicated that it is highly important (score 4.7/5) to show understanding for dementia symptoms. Concerning barriers, many participants (84%) felt that other people’s inability to cope with dementia symptoms is a significant reason for the downsizing of PWD’s social network. Moreover, the majority of the participants (52%) assumed that social stigma keeps PWD from being socially active.

**Conclusion:**

The results signify that partners and family members play an important role in initiating social experiences of PWD and in determining the benefits that result from it. Barriers seem to lay mainly in the understanding of dementia in the community, which should be addressed in future community awareness projects.

